# QSalignWeb: A Server to Predict and Analyze Protein Quaternary Structure

**DOI:** 10.3389/fmolb.2021.787510

**Published:** 2022-01-05

**Authors:** Sucharita Dey, Jaime Prilusky, Emmanuel D. Levy

**Affiliations:** ^1^ Department of Chemical and Structural Biology, Weizmann Institute of Science, Rehovot, Israel; ^2^ Department of Life Sciences and Core Facilities, Weizmann Institute of Science, Rehovot, Israel

**Keywords:** web server, protein evolution, protein quaternary structure, protein structure alignment, physiological interface, crystal contact, protein superposition, protein interactions

## Abstract

The identification of physiologically relevant quaternary structures (QSs) in crystal lattices is challenging. To predict the physiological relevance of a particular QS, QSalign searches for homologous structures in which subunits interact in the same geometry. This approach proved accurate but was limited to structures already present in the Protein Data Bank (PDB). Here, we introduce a webserver (www.QSalign.org) allowing users to submit homo-oligomeric structures of their choice to the QSalign pipeline. Given a user-uploaded structure, the sequence is extracted and used to search homologs based on sequence similarity and PFAM domain architecture. If structural conservation is detected between a homolog and the user-uploaded QS, physiological relevance is inferred. The web server also generates alternative QSs with PISA and processes them the same way as the query submitted to widen the predictions. The result page also shows representative QSs in the protein family of the query, which is informative if no QS conservation was detected or if the protein appears monomeric. These representative QSs can also serve as a starting point for homology modeling.

## Introduction

Protein self-interactions are prevalent, and they drive the formation of homo-oligomeric structures (Goodsell and Olson, 2000; Levy et al., 2005; Levy and Teichmann, 2013; Marsh and Teichmann, 2015). The spatial organization of the subunits within a protein homo-oligomer defines its quaternary structure (QS). Knowledge of the physiological QS for a protein is not only key to understand its function (Goodsell and Olson, 2000; Marianayagam et al., 2004; Amoutzias et al., 2008), but also to analyze its evolution (Franzosa and Xia, 2009; Garcia-Seisdedos et al., 2017) or to predict the impact of polymorphisms in human diseases (Yates and Sternberg, 2013).

With currently over 150,000 crystallographic structures of proteins in the Protein Data Bank (PDB) (Rose et al., 2017; Armstrong et al., 2019), much of our knowledge on protein QS comes from X-ray crystallography (Perutz et al., 1960). However, one caveat of X-ray crystallography is its requirement for protein molecules to be arranged in a regular array to form a crystal lattice. In this lattice, some protein-protein contacts may be part of a protein’s quaternary structure, whereas others only result from the crystal formation and are called crystal contacts.

Much work has been dedicated to distinguishing physiological interfaces from fortuitous crystal contacts (Capitani et al., 2016; Dey and Levy, 2018; Xu and Dunbrack, 2019; Elez et al., 2020). Several physicochemical, geometric, and evolutionary properties of the protein-protein binding surface such as amino acid composition (Ponstingl et al., 2000; Bahadur et al., 2004; Zhu et al., 2006), interface size (Janin, 1997; Henrick and Thornton, 1998; Krissinel and Henrick, 2007), shape (Tsuchiya et al., 2008), packing (Bahadur et al., 2004; Zhu et al., 2006; Tsuchiya et al., 2008), sequence conservation (Elcock and McCammon, 2001; Guharoy and Chakrabarti, 2005; Baskaran et al., 2014) or structure conservation (Xu and Dunbrack, 2011; Xu and Dunbrack, 2020) can discriminate physiological interfaces from crystal contacts. Several methods also integrated multiple features to train a classifier (Zhu et al., 2006; Bernauer et al., 2008; Mitra and Pal, 2011; Silva et al., 2015; Hu et al., 2018; Fukasawa and Tomii, 2019; Jiménez-García et al., 2019). Features range from types of atomic contacts at the interface to amino acid interface propensity scores, contact preferences, packing, co-evolution, and more.

While these works focused on individual interfaces, PQS (Henrick and Thornton, 1998) enabled predicting the full QS, which could involve more than two chains and multiple distinct interfaces (e.g., in the case of a tetramer with dihedral symmetry). Today PQS has been succeeded by PISA (Krissinel and Henrick, 2007), which predicts the stability of QSs compatible with the crystal lattice of a protein. Like PISA, EPPIC (Baskaran et al., 2014) predicts full QSs while placing emphasis on the conservation of amino acids at interfaces to infer their physiological relevance. QSalign (Dey et al., 2018; Dey and Levy, 2021) also employs evolutionary conservation, but it does not rely on sequence conservation. Instead, it searches for structural conservation of the structure of a QS across homologs. For example, if the QSs of two homologous homotetramers match, QSalign annotates both as physiologically relevant. An advantage of this approach is its accuracy: While EPPIC and PISA reach accuracies of ∼85% for homo-oligomers, the accuracy of QSalign was 96%. At the same time, a drawback of the methodology is its coverage. Homologous QSs are necessary for the annotation and as a result, only about half of the QSs in the PDB can be annotated with this strategy. Nevertheless, as more structures are solved, coverage will increase. We previously used this strategy to annotate structures from the PDB. The implementation of QSalign relied on the 3DComplex database (Levy et al., 2005), which is a classification of protein complexes of known structure. This dependency placed a barrier to generalizing this approach to any structure of choice. Here, we report the web server version of QSalign where users can upload a query structure of their choice.

## Design, Use and Performance of the QSalignWeb Server

### Server Input

The user provides two pieces of information to the server. First, an email address is required to send the results to the user. Second, a structure in PDB format is uploaded. The structure may contain a particular QS with multiple chains or may also include a single chain. The way in which the QSalign strategy works has been described and benchmarked before and we refer the reader to the original paper (Dey et al., 2018) for details.

### Server Processing

**FIGURE 1:**
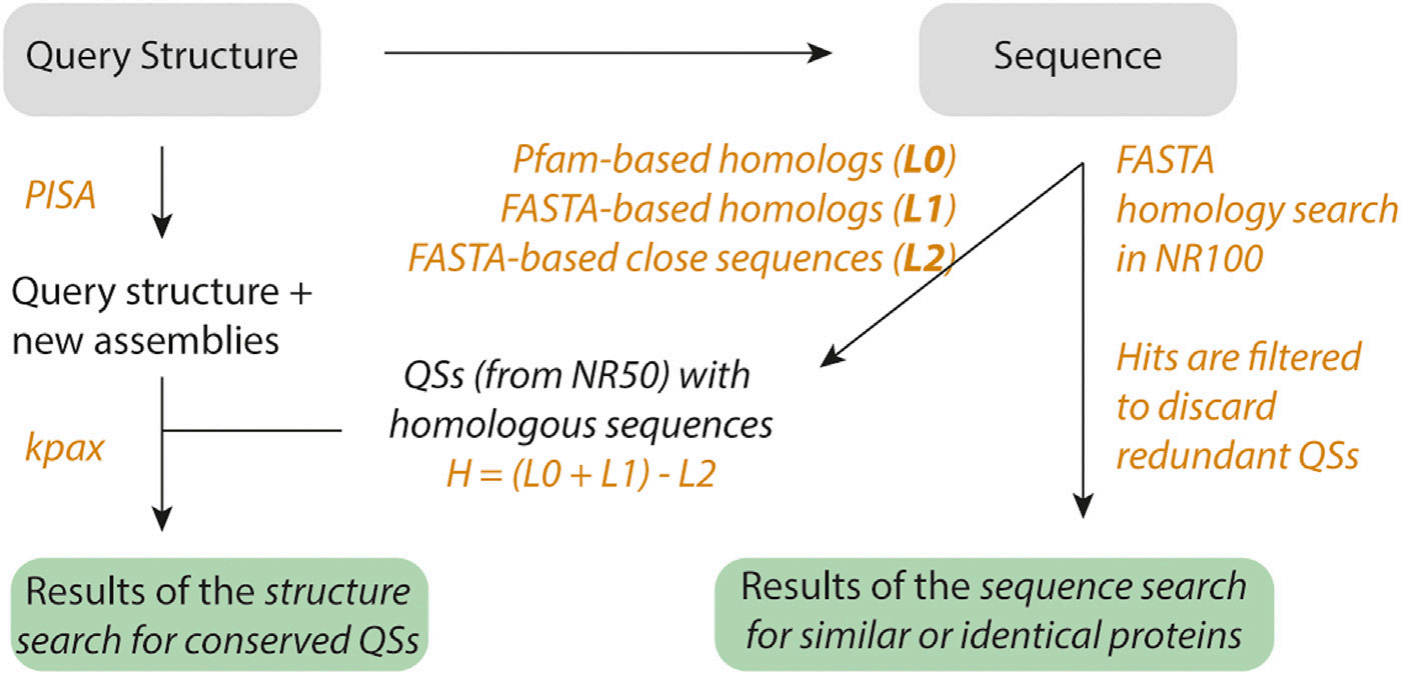
Workflow of QSalignWeb. The user submits a query structure. Additional assemblies are identified using PISA. The resulting assemblies are each superposed with candidate QSs. Because structure superposition is computationally expensive, we only superpose QSs that exhibit the same number of subunits and show sequence homology. Homologs are identified based on two searches: A sequence similarity search with FASTA yields list L1, and a PFAM domain architecture similarity search yields list L0. We take the union of these two lists, and discard very close homologs (list L2, sequence identity > 80%). The structure superposition and inference of physiological relevance is carried out as described previously (Dey et al., 2018). On top of the results of the QS superposition, we also display a table of non-redundant QSs that share sequence similarity with the query.

Upon submission of the request by the user, three main actions are performed, as described in Figure 1. First, the server analyzes the query structure to identify the number of subunits. If two or more subunits are found, the query structure is considered an assembly, referred to as A0. Then, we use PISA (Krissinel and Henrick, 2007) to generate additional possible assemblies, referred to as A1, A2, … etc. We also record the number of subunits for each assembly.

Second, the sequence of the structure is extracted, and PFAM domains are predicted based on version 33 (El-Gebali et al., 2019). The PFAM domain architecture of the query sequence is subsequently used to search for homologs with the same domain architecture. This search is executed on a non-redundant set of QSs that we call NR50 (described below). The search by domain architecture yields a list of candidate proteins called L0. Additionally, we execute a FASTA (Pearson, 1990) search on sequences from the NR50 set and retrieve two lists: L1, the list of homologs showing *less* than 80% sequence identity (this cut-off can be adjusted in the submission form) together with a sequence-coverage >70% (this cut-off can be adjusted in the submission form). We also retrieve L2, a list of structures with a sequence similar to that of the query (>80% identity). The final list of candidate QSs used in the next step results from the union of L0 and L1 after removing structures from L2. This list contains valid homologs, and we call it H. Each query assembly is then superposed using Kpax (Ritchie, 2016) onto all the QSs from list H that share the same number of subunits. If a superposition yields a TM score above 0.65 (this cut-off can be adjusted in the submission form), we infer that the query QS is conserved and likely physiological.

Third, we search for non-redundant QSs whose sequence is similar to the query sequence. This search is executed on a dataset we call NR100 (described below). The hits are then ordered by decreasing sequence identity, and only the closest target is kept per NR50 group, yielding a list of N closest distinct QS.

The NR50 and NR100 sets consist of non-redundant sets of QSs. Classically, redundancy is removed at the sequence level only. In this case, a homodimer and a monomer sharing 60% sequence identity would be grouped in the same NR50 cluster. In contrast, the non-redundant sets available in 3DComplex compare both graph topology created by connected chains along with sequence identity (Levy et al., 2005). Since a monomer and a dimer yield different graphs, they would end up in two classes. However, two homodimers can be similar at the sequence level while interacting with a different interface. In this case, they would be wrongly grouped based on their graph topology. To avoid this, we created new non-redundant sets following the procedure from 3DComplex, while also imposing that QSs in the same group show a TM-score above 0.65.

### Server Output

**FIGURE 2:**
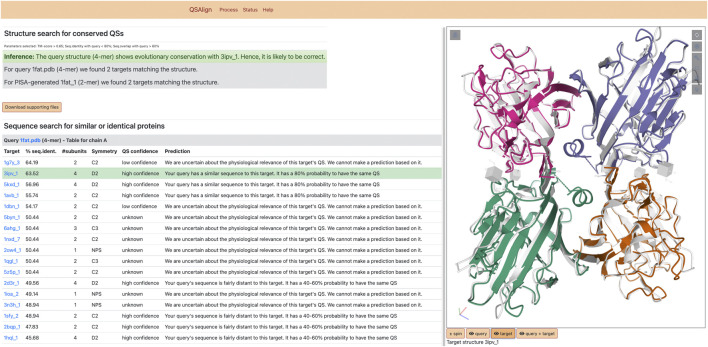
Results of QSalignWeb. The result page of QSalignWeb describes the prediction made by QSalign based on the superposition with homologous QSs. The superposition of the two QSs based on which the prediction is made is shown on the right-hand side. A table displays the result of the search of non-redundant QSs with a sequence similar to the query. In this list, the closest homolog with a high-confidence QS is highlighted in green and represents the structure we judge best for homology modeling.

The server consists of both a visual output, and a downloadable archive containing the information computed by the server. The visual output is illustrated in Figure 2. It includes an inference on the validity of the query based on the results of the QS superposition. Additionally, superposed structures are displayed using Mol* (Sehnal et al., 2021). Each QS of the superposed pair can be interactively toggled on or off to facilitate comparing their structure.

The QSs resulting from the search of the NR100 dataset are shown as a table and provide an overview of all QS types that exist among homologs, while prioritizing closely related sequences (even identical sequences). Those QSs are often annotated by QSalign, and we provide a confidence estimate for these QSs based on their QSalign annotation. The “high confidence” QS with the closest sequence to the query is highlighted and represents the best candidate for homology modeling. For example, if a user submits a single-chain structure for which no stable assembly is found with PISA, a closely related dimer annotated with high-confidence could serve as a template for modeling the query structure as a dimer.

The downloadable archive contains a list of homologs for each assembly as well as their structure in PDB format files. It also provides aligned coordinates for the query and target to enable comparing their structure using a local visualization software.

### Server Implementation

The server is hosted by the Weizmann Institute on a 64-bit machine running Linux CentOs. The backend runs on Perl and MySQL, the frontend uses Perl, JavaScript and the PDBe implementation of Mol* (https://github.com/PDBeurope/pdbe-molstar) to render the 3D representation of the molecule (Sehnal et al., 2021). QSalignWeb is freely available and requires an email address for results to be sent when they become available. For details on the QSalign methodology we refer the reader to the original paper (Dey et al., 2018).

### Server Performance

The performance of the prediction pipeline of QSalignWeb was benchmarked in previous work (Dey et al., 2018). According to this benchmark, the validation of a QS based on the conservation of its geometry yields predictions with an accuracy of 97%. Thus, the user can be confident about an inference of the server when homologs are found with a conserved QS. Also, according to the same benchmark, the correction of a QS by transitivity (i.e., query QS shows the same sequence but a different geometry to a valid QS) is more error-prone, with an accuracy of 89%.

## Conclusion

Sequence-based tertiary structure predictions have seen a recent breakthrough, notably with Alphafold2 (Cramer, 2021; Jumper et al., 2021) and Rosettafold (Baek et al., 2021). At the same time, predicting the QS of a protein remains challenging even when the tertiary structure is known. The use of evolution and coevolution information together with deep learning has also seen recent developments for scoring and predicting protein-protein interactions (Andreani et al., 2020; Quadir et al., 2021; Quignot et al., 2021; Yan and Huang, 2021). Complementary to such residue-level information, the use of subunit interaction geometry conservation as evidence of a QS being physiological is a powerful approach, which yields accurate predictions (Dey et al., 2018; Dey and Levy, 2021). In that respect, we hope that making this prediction pipeline available as a web server will help biologists identify relevant QS of proteins and will help them investigate the QS of specific proteins. Together, these approaches will help bridge the gap between the sequence and structure space by adding a third dimension to proteomes and interactomes (Aloy and Russell, 2006; Rolland et al., 2014; Elofsson, 2021; Postic et al., 2021; Sali, 2021), thus making 3D proteomics or “structuromics” (Levy and Vogel, 2021) accessible.

## Data Availability

The original contributions presented in the study are included in the article/Supplementary Material, further inquiries can be directed to the corresponding author.
